# *Cryptococcus neoformans* Thermotolerance to Avian Body Temperature Is Sufficient For Extracellular Growth But Not Intracellular Survival In Macrophages

**DOI:** 10.1038/srep20977

**Published:** 2016-02-17

**Authors:** Simon A. Johnston, Kerstin Voelz, Robin C. May

**Affiliations:** 1 Department of Infection, Immunity and Cardiovascular Disease, Medical School, University of Sheffield, Sheffield, UK; 2 Bateson Centre, University of Sheffield, Sheffield, UK; 3 Institute of Microbiology and Infection and School of Biosciences, University of Birmingham, Birmingham, UK; 4 NIHR Surgical Reconstruction and Microbiology Research Centre, University Hospitals of Birmingham NHS Foundation Trust, Queen Elizabeth Hospital, Birmingham, UK

## Abstract

*Cryptococcus neoformans* is a fatal fungal pathogen of humans that efficiently parasitises macrophages. Birds can be colonised by cryptococci and can transmit cryptococcosis to humans via inhalation of inoculated bird excreta. However, colonisation of birds appears to occur in the absence of symptomatic infection. Here, using a pure population of primary bird macrophages, we demonstrate a mechanism for this relationship. We find that bird macrophages are able to suppress the growth of cryptococci seen in mammalian cells despite *C. neoformans* being able to grow at bird body temperature, and are able to escape from bird macrophages by vomocytosis. A small subset of cryptococci are able to adapt to the inhibitory intracellular environment of bird macrophages, exhibiting a large cell phenotype that rescues growth suppression. Thus, restriction of intracellular growth combined with survival at bird body temperature explains the ability of birds to efficiently spread *C. neoformans* in the environment whilst avoiding systemic disease.

*Cryptococcus neoformans* is an environmental fungus that causes fatal human and animal disease. In humans, cryptococcosis causes hundreds of thousands of deaths each year, the vast majority in immunocompromised patients^1^. As with many significant pathogens, cryptococci are able to parasitise host cells. This potential for an intracellular lifestyle allows *C. neoformans* the potential to evade additional host immune responses and thus spread within the body, leading to systemic disease^2^,^3^.

*Cryptococcus* has a remarkably broad host range but with significant variation in susceptibility between species in both natural and experimentally induced infection^4^. Birds are particularly resistant to disease, despite diverse avian orders (Passeriformes, Anseriformes, Accipitriformes, Apterygiformes, Psittaciformes and Columbiformes) showing clear environmental association with *Cryptococcus* and the ability to transmit the pathogen to humans^5^,^6^,^7^,^8^,^9^,^10^,^11^. The exposure of birds to cryptococci in the environment will result in carriage externally on e.g. beaks and claws, but also internal carriage following the ingestion of contaminated vegetable matter or while ground feeding^12^,^13^,^14^,^15^,^16^,^17^. Bird guano is an excellent growth medium for *Cryptococcus* and external carriage of cryptococci by birds is a likely route for inoculation along with air dispersion of spores. However, if cryptococci are able to survive passage through birds this would provide a direct route for inoculation. Transmission of this pathogen from birds to humans has important implications both for public health (e.g. exposure of immunocompromised individuals to urban pigeons and guano) and for the global dispersal of specific fungal lineages^18^,^19^,^20^. The underlying mechanism of avian resistance to cryptococcosis is unknown but has been hypothesised to be reliant on higher body temperature^21^. Here we test this hypothesis directly and show, surprisingly, that high body temperature alone is insufficient to prevent cryptococcal growth. However, at avian body temperatures, bird macrophages strongly suppress fungal growth but a small proportion of the fungal cell population escape killing. Thus, we suggest a model whereby birds can harbour low numbers of cryptococci for prolonged periods without disease and are therefore able to act as vectors of long-range fungal carriage and are able to directly inoculate guano^22^.

## Results

### Avian macrophages suppress intracellular growth of cryptococci

**Figure 1:**
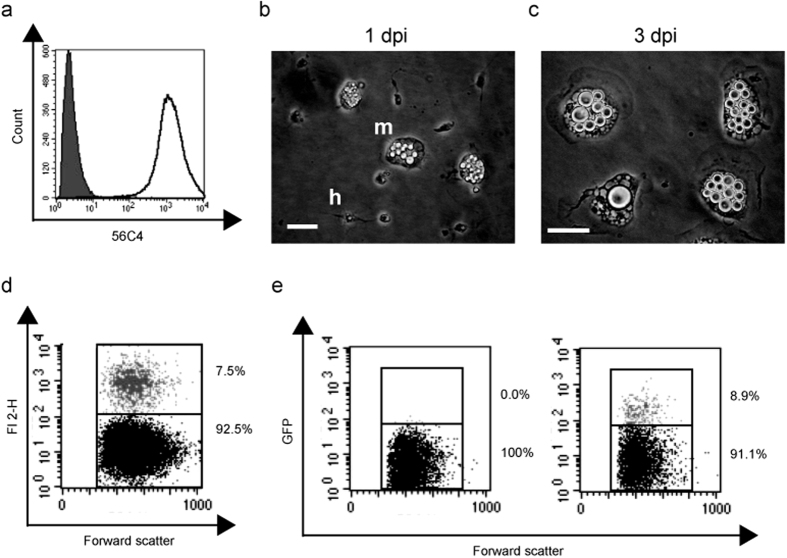
Isolation and characterisation of monocyte derived macrophages from chicken peripheral blood. (**a**) Representative plot of cell population isolated from chicken peripheral blood labeled with monocyte/macrophage marker (KUL01; unfilled) in comparison to isotype control labeling (filled). (**b**) Adherent isolated cells 1 day post isolation (dpi). m, monocyte and h, heterophil. Scale bar 20 μm. (**c**) Monocyte derived macrophages 3 days post isolation. Scale bar 20 μm. (**d**) Phagocytosis of red fluorescent latex beads measured by flow cytometry. (**e**) Phagocytosis of H99GFP cryptococci measured by flow cytometry. Left panel macrophages alone, right panel macrophages plus H99GFP. Gray dots represent cells containing cryptococci (black dots represent macrophages without intracellular cryptococci).

Given the importance of macrophage parasitism in mammalian cryptococcal infection, we established a primary avian macrophage model to investigate *Cryptococcus* virulence in birds. To achieve this, we generated a highly purified population of primary, monocyte derived, macrophages from chicken peripheral blood (Fig. 1a). One day post-isolation, adherent cells were a mixed population of monocytes and heterophils (Fig. 1b). After 48 hours heterophils were lost and monocyte derived macrophages (MDMs) remained (Fig. 1c). Avian macrophages efficiently phagocytosed both cryptococci and latex beads of the same diameter (Fig. 1d,e).

**Figure 2:**
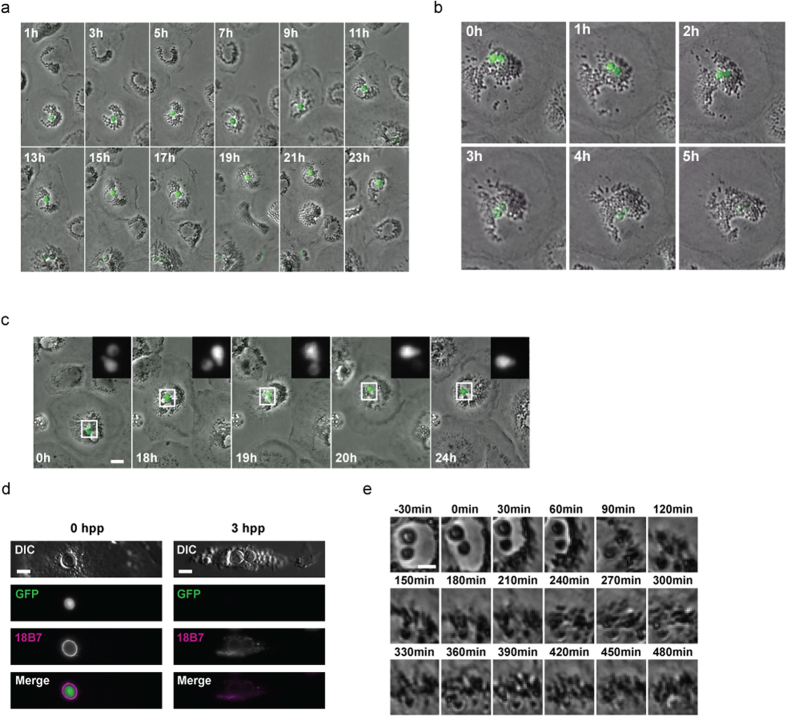
Resistance of avian macrophages to parasitism by *Cryptococcus*. (**a**) Absence of cryptococcal intracellular proliferation in avian macrophages. Phase contrast and fluorescent time lapse microscopy of intracellular GFP expressing cryptococcal strain H99GFP. Images representative of 50 cells from n = 3 experiments. Scale bar 10 μm. (**b**) Intracellular killing of cryptococci in primary avian macrophage shown by degradation of GFP signal (green) over time. Phase contrast and fluorescent time-lapse microscopy of intracellular GFP expressing cryptococcal strain H99GFP. Images representative of 57 cells from n = 3 experiments. Scale bar 10 μm. (**c**) Loss of GFP fluorescence from one of two intracellular cryptococci. Phase contrast and fluorescent time lapse microscopy of intracellular GFP expressing cryptococcal strain H99GFP over 24 hours. (**d**,**e**) Degradation of cryptococci accompanies loss of GFP signal. (**d**) Immunofluoresence labeling of capsular polysaccharide, a cryptococcal virulence factor, 0 and 3 hours post phagocytosis (hpp). Loss of GFP signal at 3 hpp is associated with degradation of capsule and production of macrophage vesicles containing capsular polysaccharide. Differential interference contrast (DIC) and fluorescence microscopy of fixed cells. Scale bar 5 μm. (**e**) Degradation of intracellular cryptococci following loss of GFP. Phase contrast time-lapse microscopy. Time = 0 indicates loss of visible GFP signal. Scale bar 5 μm.

In contrast to mammalian macrophages, intracellular proliferation of cryptococci within avian macrophages was extremely rare. Instead intracellular cryptococci appeared inert (Fig. 2a) or were killed (observed via loss of cytoplasmic GFP signal (Fig. 2b) that was independent of any photobleaching (Fig. 2c)). Loss of GFP signal occurred with degradation and dispersal of polysaccharide capsule (Fig. 2d) and subsequent fungal cell digestion (Fig. 2e).

### Avian body temperature is necessary but not sufficient to prevent cryptococcal growth

**Figure 3:**
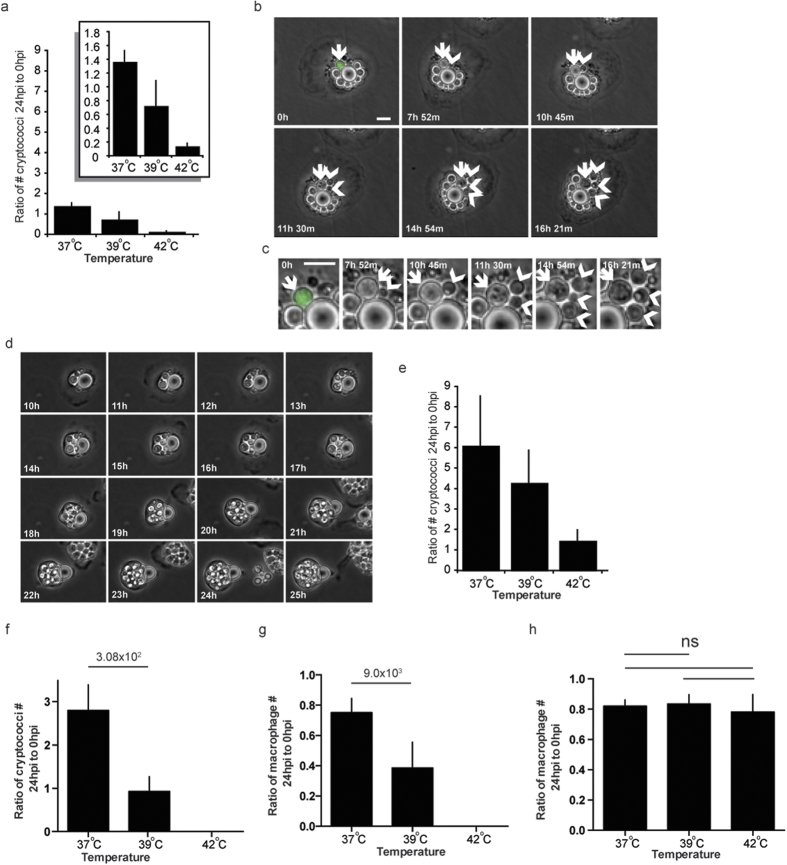
Temperature modulates parasitism of avian macrophages by *Cryptococcus*. (**a**) Number of cryptococci in avian macrophages after 24 hours as a ratio to 0 hours post phagocytosis. Identical scale as (**e**), insert panel Intracellular proliferation values plotted on larger scale. (**b,c,d**) *Cryptococcus* intracellular proliferation in avian macrophages at 37 °C (**b**) Sequential individual budding events from single mother cell in avian macrophages at 37 °C. Arrow, mother cell. Arrow heads, daughter cells. (**c**) Enlargement of arrowed cells from (**b**). (**d**) Massive intracellular proliferation over 15 hours at 37 °C. Scale bars 10 μm. (**e**) Number of cryptococci growing extracellularly after 24 hours as a ratio to 0 hours. P-values for comparison of Intracellular growth in avian macrophages (I) and Extracellular growth (E): Intracellular (I) 37 and 42 °C n = 9; (I) 39 °C n = 6; Extracellular (E) n = 3. I37 vs. I39 P = 5.6 × 10^−3^; I37 versus I42 P = 3.5 × 10^−4^; I37 vs E42 P = 1.5 × 10^−3^. I39 vs, I42 P = 1.310^−2^; I42 vs. E42 P = 1.3 × 10^−3^; E37 vs. E42 P = 4.9 × 10^−2^; E39 vs E42 P = 4, 9 × 10^−2^. (**f**) Number of cryptococci in murine macrophages after 24 hours as a ratio to 0 hours post phagocytosis. (**g**) Number of murine macrophages after 24 hours as a ratio to 0 hours post phagocytosis of cryptococci. (**h**) Number of avian macrophages after 24 hours as a ratio to 0 hours post phagocytosis of cryptococci. Error bars are standard deviation of the mean.

Quantitation of viable intracellular yeast 24 hours post phagocytosis confirmed our time-lapse data, indicating that there was a greater than 80% reduction in the numbers of cryptococci in avian macrophages at a typical bird body temperature of 42 °C (Fig. 3a). However, a shift in temperature from 42 °C to 39 °C resulted in a restoration in the number of cryptococci and a shift to 37 °C reinstated the ability of cryptococci to proliferate in macrophages (Fig. 3a and see Supplementary Movie S1 online). Budding cryptococci could be clearly observed within avian macrophages at 37 °C by microscopy (Fig. 3b,c), with 70% of macrophages exhibiting large phagosomes containing many cryptococci after 24 hours (Fig. 3d). To compare the direct effect of temperature on cryptococcal growth we measured growth of extracellular cryptococci (i.e. growth in macrophage media). We found that, while the number of cryptococci was similarly modulated by temperature, there was still an increase in cryptococcal numbers over 24 hours at 42 °C in contrast to the 80% reduction (Fig. 3e) at the same temperature in bird macrophages. Thus temperature alone cannot explain this restriction of cryptococcal growth, but rather the enhanced activity of avian macrophages at the bird body temperature of 42 °C degrees is responsible for preventing expansion of the intracellular pathogen population.

Mammals are typically unable to sustain core temperatures of 42 °C for prolonged periods. Nonetheless, we tested the ability of *Cryptococcus* to grow in murine macrophages above 37 °C. Incubation of murine macrophages at 39 °C saw a large reduction in numbers of cryptococci present at 24 hours post phagocytosis but at 42 °C zero cryptococci were present (Fig. 3f). However, murine macrophage numbers were significantly reduced at 39 °C, and completely ablated at 42 °C (Fig. 3g), suggesting that reduced numbers of cryptococci measured was due to loss of macrophages from the assay and not increased cryptococcal killing, a loss that does not occur with avian cells (Fig. 3h).

### Vomocytosis is conserved as a mechanism of escape for *Cryptococcus* in avian macrophages irrespective of successful parasitism

**Figure 4:**
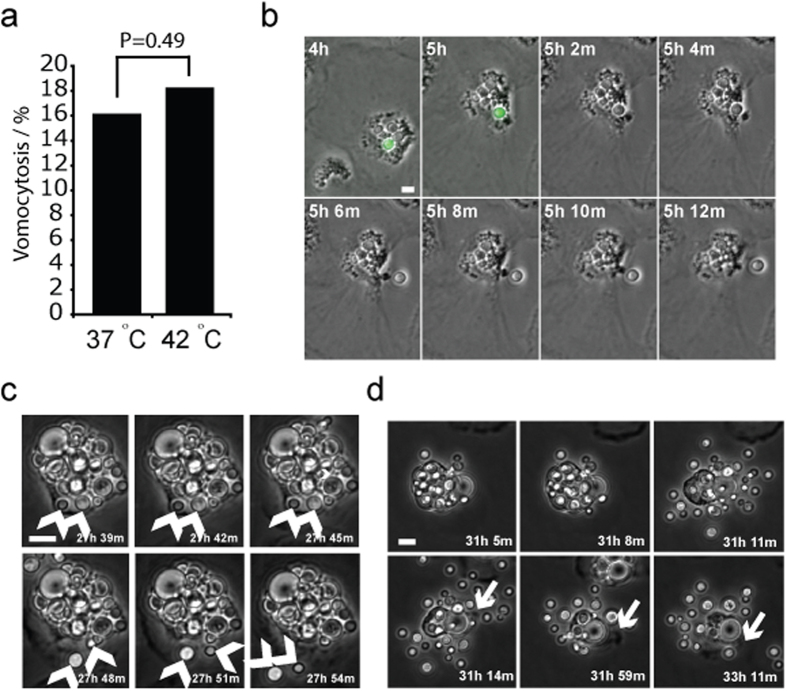
Escape from avian macrophages by vomocytosis. (**a**) Quantification of vomocytosis over 24 hours from time lapse microscopy. Pooled data from n = 3 experimental repeats. (**b**) Example of vomocytosis at 42 °C from Supplementary Movie S2 online. Arrow indicates cryptococcal cell. (**c**) Example of single vomocytic event at 37 °C from Supplementary Movie S3 online. Arrows indicate cryptococci before and after vomocytosis. (**d**) Example of large vacuole vomocytosis at 37 °C from Supplementary Movie S4 online. Arrow indicates ruffling of macrophage pseudopod post vomocytosis showing that cell is entirely viable post expulsion. Scale bars 10 μm.

Vomocytosis is the non-lytic escape of *Cryptococcus* from host cells^8^,^10^. Given the severe defect in *Cryptococcus* host cell parasitism in avian macrophages we hypothesized that vomocytosis would be similarly affected. However, cryptococci were efficiently vomocytosed from avian macrophages at 42 °C and at an incidence comparable to that measured in mammalian macrophages^23^ at 37 °C (Fig. 4a,b and Supplementary Movie S2 online). Similarly, the incidence of vomocytosis was not affected by shifting avian macrophages to 37 °C (Fig. 4a) despite a dramatic change in phagosome morphology that resulted in a combination of multiple single expulsion events^8^ (Fig. 4c and Supplementary Movie S3 online) and massive vacuole exit^10^ (Fig. 4d and Supplementary Movie S4 online). Taken together these data demonstrate both that vomocytosis is conserved in birds and that cryptococci can escape host cells under unfavourable conditions.

### Higher host cell temperature drives protective increase in fungal cell size

**Figure 5:**
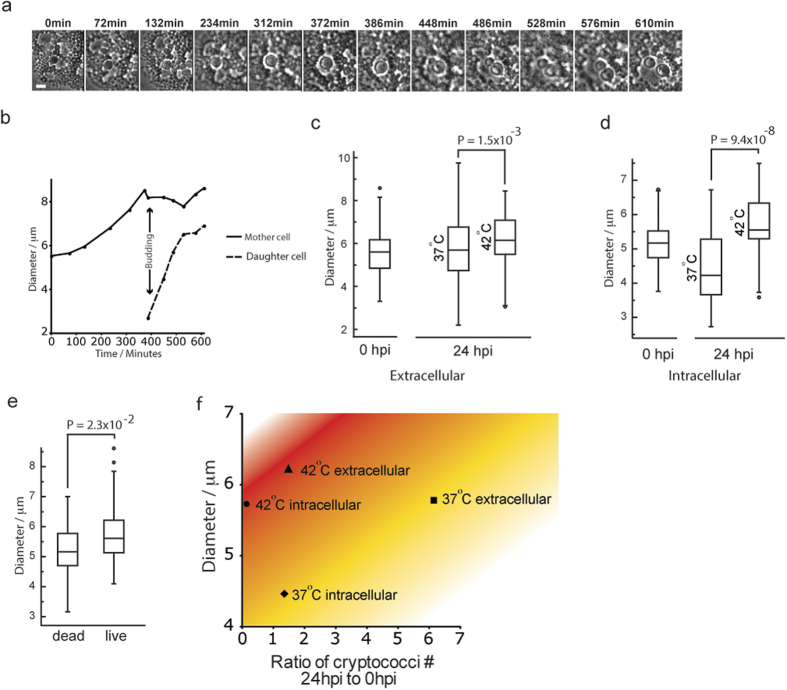
Increased cell size restores proliferation of a subset of cryptococci in avian macrophages at 42 °C. (**a**) Example of increase in cell size and cell proliferation of small proportion of intracellular cryptococci. Phase contrast time-lapse microscopy. Scale bar 5 μm. (**b**) Quantitation of cryptococcal cell diameter during enlargement and budding phases. Normal range of cryptococcal cell diameter is 3–5 μm. Same cell as shown in (**a**). (**c**) Cell diameter of intracellular cryptococci at 0 and 24 hpp from n = 3 experiments at 37 °C and 42 °C. (**d**) Increase in extracellular cryptococcal cell size in response to temperature. Cell diameter of extracellular cryptococci at 0 and 24 hpp from n = 3 experiments at 37 °C and 42 °C. (**e**) Cell diameter of live and dead cryptococci. Box plots drawn with line = median, box = interquartile range, bar = 5–95%, dots=outlier. Measurements were made at identical time points 1 hour prior to loss of GFP fluorescence of dead cell. Equivalent live cell was selected randomly from same field of view. 80 cells from n = 3 experiments. (**f**) Chart of the relationship between cryptococcal cell size, growth, location and temperature. Cryptococcal number ratios are taken from means plotted in Fig. 3a,e and cell diameter are means from data plotted in (**c,d**).

Careful analysis of time-lapse experiments showed that a small subset of intracellular cryptococci became enlarged, rather than exiting via vomocytosis^11^ and this increase in cell size was sufficient to restore some level of proliferative ability (6% over 24 hours, pooled data from 131 cells from n = 3 experiments; Fig. 5a,b; see Supplementary Movie S5 online). After 24 hours extracellular growth at 37 °C, cryptococci showed no change in cell size compared to the initial population (Fig. 5c; P = 0.12), while intracellular growth in avian macrophages at 37 °C resulted in a reduction in cell size in comparison to the initial population (Fig. 5d; P = 2.6 × 10^−6^). In contrast, both intracellular and extracellular cryptococci showed significant increases in size over 24 hours at 42 °C (Fig. 5c,d). We therefore considered that this cell size increase might be protective, permitting survival of cryptococci. In support of this hypothesis, measurement of cryptococcal cell size from identical time points in time-lapse experiments demonstrated that larger cells were significantly more likely to survive within avian macrophages (Fig. 5e). Thus the higher avian body temperature is necessary for the innate control of cryptococcal infection but also induces a protective increase in fungal cell size that allows the pathogen to persist (Fig. 5f).

## Discussion

Here we show that primary avian macrophages, in contrast to those from mammals, suppress growth of the fatal fungal pathogen *Cryptococcus neoformans*. Avian macrophages alone were able to kill the majority of internalized cryptococci but this activity is dependent on the higher avian body temperature. Mammalian macrophages appeared to similarly suppress growth of cryptococci but were found to be killed at 39 °C and 42 °C. Cryptococci are able to escape avian cells via vomocytosis and respond to the higher host temperature by means of a protective cell enlargement. Such survival methods highlight the success of *Cryptococcus* as an environmental opportunistic pathogen, which can exploit a diverse range of host species.

Intracellular growth of cryptococci in macrophages is one of the earliest described characteristics of human cryptococcal cellular pathogenesis^24^, correlates with disease and is conserved in such diverse organisms as insect phagocytes^25^ and social amoebae^26^. What then determines the resistance of avian macrophages to cryptococcal proliferation? Tolerance of relatively high body temperatures is the primary determinant of fungal infections in homoeothermic hosts and explains why so few fungi are pathogens of mammals^21^. However, *Cryptococcus neoformans* is a remarkably thermotolerant yeast^27^ and this thermotolerance is the primary distinguishing feature for disease-causing cryptococcal species, even in the presence of other major virulence factors. The maximal growth range of *Cryptococcus* varies between strains and by growth environment but shows significant overlap with avian body temperature^28^. In agreement with this we show that *Cryptococcus* is able to grow at 42 °C in an extracellular niche. However, cryptococci were unable to grow intracellularly in avian macrophages, and shifting avian macrophages to lower temperatures alone restored the intracellular proliferative capacity of cryptococci, thus demonstrating the essential contribution of this innate cellular immune response to suppression of cryptococcosis in birds.

Remarkably, we also demonstrate that vomocytosis of intracellular cryptococci occurs in avian macrophages in a way that is indistinguishable from mammalian vomocytosis in either morphology or frequency. In addition, this process appears to be unaffected by temperature of the avian cell and when proliferation is severely restricted. The cause and function of vomocytosis during animal infection is still unclear but vomocytosis offers a clear advantage in the avoidance of predation by soil amoebae predators^29^ and these data support the fundamental importance and conservation of this unusual phenomenon.

We identified two distinct cryptococcal cell size changes during interaction with avian macrophages. The reduction in cell size in comparison to the initial population observed in intracellular cryptococci at 37 °C demonstrated the sensing of a lack of nutrient resources, resulting in low growth rate and small cell size^30^. In contrast, extracellular cryptococci showed no difference in cell size, and a good growth rate, at 37 °C. Very little proliferation occurred intracellularly at 42 °C but those cells that did proliferate first significantly increased their cell size (6% of intracellular population, pooled data from 131 infected macrophages from n = 3 experiments). This increase in size was smaller than that exhibited by titan/giant cryptococcal cells found during mammalian infections^31^,^32^ but is more similar to the changes seen in mutant cells lacking phospholipase b1 activity (*plb1*) in mammalian macrophages^33^. Such sub-population changes are also similar to the ‘division of labour’ phenotype described for *C. gattii*^34^. In agreement with our data increased cell size appears to be driven by adaptation to host stress^31^.

In conclusion, effective phagocyte function at the higher avian body temperature results in almost total suppression of cryptococcal proliferation within bird macrophages. However, a minority of cryptococci are able to escape killing, either by a protective cell enlargement or via vomocytosis. While it is likely that external carriage, e.g. on beaks or claws, is sufficient for the inoculation of guano and other environmental we are able to demonstrate that internal carriage is possible, and even likely, following any ingestion of cryptococci. This provides a cellular basis for the presentation and outcome of avian cryptococcosis, for asymptomatic internal carriage and for localised infections of low-temperature tissues to occur (e.g. cutaneous infection) while systemic disease is extremely rare.

## Materials and Methods

### *Cryptococcus* Culture and infection

Cryptococcal strain H99GFP^23^ was grown for 18 hours in YPD (2% glucose, 1% peptone, and 1% yeast extract) rotating (20 rpm) at 25 °C. Yeast cells were pelleted from culture by centrifugation (2 minutes, 4000 g) and resuspended in PBS.

### Isolation of Primary Chicken Monocyte Derived Macrophages

Chicken monocytes were isolated from chicken peripheral blood (Firstlink, Birmingham, UK). Blood was diluted 1:1 with sterile PBS, layered onto a Ficoll Plaque Plus (GE Healthcare, Vienna) cushion at a ratio of 1:1 and centrifuged at 285 × g for 30 minutes with no braking. Cells were harvested from the Ficoll interface, diluted 1:1 with sterile PBS and pelleted by centrifugation at 200xg for 5 minutes, washed once in PBS and resuspended in RPMI with 5% chicken serum, 50 U/ml penicillin and 50 μg/ml streptomycin. Cell suspension was diluted to 1 × 10^6^ cells per ml and 20ml plated per 75 cm^2^ tissue culture flask at 42 °C in a humidified atmosphere of 5% CO_2_. After 24 hours non-adherent cells were removed. Day 1 post isolation adherent cells were a mixed population of monocytes and heterophils (Fig. 1b). After 48 hours adherence to plastic, heterophils were lost and monocyte derived macrophages (MDMs) remained^35^. MDMs showed characteristic flattening and increased cell size (Fig. 1c). MDMs were detached with Accutase treatment (PAA), counted and 1ml of a 2 × 10^5^ cells per ml solution plated in 24-well plates. Purity of cell population was measured, after each isolation, by flow cytometry (FACSCaliber and CellQuestPro software, BD Biosciences) after labelling with PE conjugated mAb 56C4 IgG1 (Southern Biotech) or isotype control (Fig. 1a).

### Phagocytosis of *Cryptococcus* and Latex Beads

Cryptococci or red fluorescent latex beads (L3030; 2 μm mean particle size; Sigma, Poole, UK) were resuspended in RPMI, added to chicken macrophages at a 10:1 ratio and incubated for 2 hours at 42 °C in a humidified atmosphere of 5% CO_2_. Phagocytosis efficiency was measured by flow cytometry (FACSCaliber and CellQuestPro software, BD Biosciences).

### Live Imaging

After phagocytosis of cryptococci, chicken primary macrophages were washed at least five times in PBS before imaging. Time lapse images were captured on a TE2000 inverted microscope (Nikon) with Digital Sight DS-Qi1MC camera (Nikon), 20× objective (Ph1 PLAN APO), using NIS elements AR software (Nikon). Phase contrast images were captured every 2 minutes and fluorescence images were captured every hour for between 24 and 48 hours. The microscope was enclosed in a temperature controlled and humidified environmental chamber (Okolabs) with 5% CO_2_ at either 37 °C or 42 °C.

### Labelling and imaging of fixed cells

Cells were fixed for 10 minutes in 4% formaldehyde in PBS. TRITC-phalloidin labelling of actin and immunocytochemistry was performed as described previously^36^. Antibody to cryptococcal capsule (mAb 18B7; gift from Arturo Casadevall) was used at 1 μg/ml. Images were captured on TE2000 (Nikon) with 12-bit QICAM (QImaging), 60× objective (CFI Plan Apo TIRF oil 1.49NA) using NIS elements AR software (Nikon).

### Quantification of Intracellular and Extracellular Proliferation

For quantification of intracellular growth at 37 °C, 39 °C or 42 °C, each well was washed with PBS at least five times, to remove unphagocytosed extracellular yeast cells, and 1 ml RPMI was added. At each time point (0 h, 18 h, 24 h and 48 h) media was removed and 200 μL sterile dH2O was added to lyse macrophage cells. After 30 min, the intracellular yeast were released and collected. Another 200 μL dH2O was added to each well to collect the remaining yeast cells. We have previously shown this approach does not adversely affect yeast viability (Ma *et al*. 2009). The yeast cell suspension was diluted and plated on YPD agar for colony forming unit (cfu) counting. This approach was performed in parallel with haemocytomer and flow cytometry measurement as described previously^23^,^37^,^38^ but these approaches proved less accurate due to inability to distinguish cryptococci from macrophage vesicles in the case of haemocytometer counting and GFP fluorescent dead cryptococci from live cells with flow cytometry. The numbers of cryptococci presented are the ratio of the number of cryptococci at each time point divided by the number immediately after phagocytosis. For quantification of extracellular growth, RPMI media alone was inoculated identically to macrophages, incubated at 37 °C, 39 °C or 42 °C and diluting cfu counts were taken at each time point.

### Measurement of cryptococcal cell size

Diameters of intracellular and extracellular cryptococci were calculated from measurements of cell area in ImageJ from images of fixed samples prepared as described above. For comparison of live and dead cells in time lapse experiments cell area was measured in NIS elements AR (Nikon) in the frame immediately following that in which fluorescence loss was observed in a dead cell and a corresponding live cell from the same frame and field of view.

### Image processing

TE2000 images from NIS elements AR were exported as individual tiff files and transformed into tiff stacks using ImageJ. All Quicktime movies were made using ImageJ with Mpeg4 compression. Individual images for figures were copied from ImageJ into Photoshop CS3 (Adobe), which was then used to form RGB merges and to adjust contrast. Illustrator CS3 (Adobe) was used to assemble figures and add scale bars, time indexes, arrows etc. except where noted in figure legend.

### Statistical analysis

Statistical significance was calculated using the Fisher exact test add-in (http://www.obertfamily.com/software/fisherexact.html) in Microsoft Excel for pooled categorical data and using a Mann-Whitney U-test (http://elegans.swmed.edu/leon/stats/utest.html; Leon Avery) for pooled continuous data. Box plots were generated using http://www.physics.csbsju.edu/cgi-bin/stats/anova_pnp_form.sh?ngroup=n (line = median, box = interquartile range (IQR), bar = 5–95%, dots = outlier).

## Additional Information

**How to cite this article**: Johnston, S. A. *et al*. *Cryptococcus neoformans* Thermotolerance to Avian BodyTemperature Is Sufficient For Extracellular Growth But NotIntracellular Survival In Macrophages. *Sci. Rep*. **6**, 20977; doi: 10.1038/srep20977 (2016).

## Supplementary Material

Supplementary Information

Supplementary Movie S1

Supplementary Movie S2

Supplementary Movie S3

Supplementary Movie S4

Supplementary Movie S5
