# Supplementary material for: Cryptococcus neoformans Thermotolerance to Avian Body Temperature Is Sufficient For Extracellular Growth But Not Intracellular Survival In Macrophages
Source: Sci Rep. 2016 Feb 17;6:20977. doi: 10.1038/srep20977 (PMC4756366; doi:10.1038/srep20977)
Supplement: Supplementary Information [file srep20977-s1.pdf]

Cryptococcus neoformans Thermotolerance to Avian Body Temperature Is Sufficient For Extracellular Growth But Not Intracellular Survival In Macrophages

Simon A Johnston, Kerstin Voelz, Robin C May

Supplementary Movie 1. Phase contrast microscopy time lapse movie of intracellular proliferation of cryptococci in avian macrophages at 37°C shown in Fig. 3b,c. Movie is displayed at 2 frames per second.

Supplementary Movie 2. Phase contrast and fluorescence microscopy time lapse showing example of vomocytosis in an avian macrophage at 42°C. First 10 frames are still images merged to show location of cryptococcal cell (GFP; green) within macrophage. Each frame is 2 minutes apart and movie is displayed at 15 frames per second.

Supplementary Movie 3. Phase contrast microscopy time lapse showing an example of three individual vomocytic events from a single avian macrophage at 37°C. Each frame is 2 minutes apart and movie is displayed at 25 frames per second. Arrows indicate location of next vomocytosis. First vomocytic event is shown in Fig. 4c.

Supplementary Movie 4. Phase contrast microscopy time lapse showing an example of a massive vomocytic event from an avian macrophage at 37°C. Each frame is 2 minutes apart and movie is displayed at 5 frames per second.

Supplementary Movie 5. Merged phase contrast and fluorescence microscopy time lapse showing intracellular fate of three cryptococcal cells (GFP; green), a, is killed and degraded, b, is unchanged, c, enlarges significantly and buds twice (c’ and c’’). Region of interest around (c) is shown in Fig. 5a. Each frame is 1 hour apart and movie is displayed at 2 frames per second.
